# Translational evaluation of novel selective orexin-1 receptor antagonist JNJ-61393215 in an experimental model for panic in rodents and humans

**DOI:** 10.1038/s41398-020-00937-9

**Published:** 2020-09-07

**Authors:** Giacomo Salvadore, Pascal Bonaventure, Anantha Shekhar, Philip L. Johnson, Brian Lord, Brock T. Shireman, Terry P. Lebold, Diane Nepomuceno, Christine Dugovic, Sander Brooks, Rob Zuiker, Cathy Bleys, Kanaka Tatikola, Bart Remmerie, Gabriel E. Jacobs, Koen Schruers, John Moyer, Abigail Nash, Luc G. M. Van Nueten, Wayne C. Drevets

**Affiliations:** 1 Janssen Research & Development, LLC, Titusville, NJ USA; 2 Janssen Research & Development, LLC, San Diego, CA USA; 3 Departments of Psychiatry, and Pharmacology, Indiana University, School of Medicine, Indianapolis, IN USA; 4 Department of Anatomy, Physiology and Cell Biology, Indiana University, School of Medicine, Indianapolis, IN USA; 5 Centre for Human Drug Research, Leiden, The Netherlands; 6 Leiden University Medical Center, Leiden, The Netherlands; 7 Janssen Research & Development, LLC, Beerse, Belgium; 8 Janssen Scientific Affairs, LLC, Titusville, NJ USA; 9 Department of Psychiatry, Leiden University Medical Center, Leiden, The Netherlands; 10 Research School for Mental Health and Neuroscience, Maastricht University, Maastricht, The Netherlands

**Keywords:** Pharmacodynamics, Physiology

## Abstract

Orexin neurons originating in the perifornical and lateral hypothalamic area project to anxiety- and panic-associated neural circuitry, and are highly reactive to anxiogenic stimuli. Preclinical evidence suggests that the orexin system, and particularly the orexin-1 receptor (OX1R), may be involved in the pathophysiology of panic and anxiety. Selective OX1R antagonists thus may constitute a potential new treatment strategy for panic- and anxiety-related disorders. Here, we characterized a novel selective OX1R antagonist, JNJ-61393215, and determined its affinity and potency for human and rat OX1R in vitro. We also evaluated the safety, pharmacokinetic, and pharmacodynamic properties of JNJ-61393215 in first-in-human single- and multiple-ascending dose studies conducted. Finally, the potential anxiolytic effects of JNJ-61393215 were evaluated both in rats and in healthy men using 35% CO_2_ inhalation challenge to induce panic symptoms. In the rat CO_2_ model of panic anxiety, JNJ-61393215 demonstrated dose-dependent attenuation of CO_2_-induced panic-like behavior without altering baseline locomotor or autonomic activity, and had minimal effect on spontaneous sleep. In phase-1 human studies, JNJ-61393215 at 90 mg demonstrated significant reduction (*P* < 0.02) in CO_2_-induced fear and anxiety symptoms that were comparable to those obtained using alprazolam. The most frequently reported adverse events were somnolence and headache, and all events were mild in severity. These results support the safety, tolerability, and anxiolytic effects of JNJ-61393215, and validate CO_2_ exposure as a translational cross-species experimental model to evaluate the therapeutic potential of novel anxiolytic drugs.

## Introduction

Orexin neuropeptides, also known as hypocretins, are critically involved in coordinating adaptive physiological, behavioral, and endocrine responses to salient stimuli or conditions, including stress and exposure to aversive or threatening stimuli. The orexin cell bodies are restricted to the tuberal hypothalamus, which plays key roles in regulating multiple physiological functions^1,2^. In addition to projections to other hypothalamic and brainstem nuclei, and to cortical and subcortical structures associated with medial prefrontal cortical network, the extensive distribution of axonal projections from orexin neurons to the central nucleus of the amygdala and the bed nucleus of the stria terminalis putatively supports the synchronization and amplification of their firing activity in anxiety, fear, and motivated states^3^.

At the terminals of these projections, orexin interacts with two distinct receptors, orexin-1 (OX1R) and orexin-2 receptor (OX2R) subtypes. Preclinical studies have provided insights into the mechanism of orexin system as a wakefulness stabilizer in normal sleep–wake cycle^4^, and have shown that antagonism of OX2R is sufficient to induce and prolong sleep in rodents^5^ and humans^6^. The key role of the orexin system in sleep/wakefulness regulation is characterized by the specific anatomical distribution of OX2Rs in histaminergic neurons in the tuberomammillary nucleus^7^. In contrast, OX1Rs are more selectively expressed in bed nucleus of the stria terminalis, amygdala, cingulate cortex, and the noradrenergic neurons of the locus coeruleus^8^. Consistent with the anatomical distribution of OX1R, a critical role for this receptor is emerging in complex emotional behavior, such as association of OX1R pathway overactivation with panic or anxiety states^9–13^.

Orexin neurons originating in the perifornical and lateral hypothalamic area are highly reactive to anxiogenic stimuli, and have strong projections to anxiety- and panic-associated circuitry expressing OX1R^13,14^. In rats, after administration of the anxiogenic inverse benzodiazepine agonist FG-7142^15^, a selective OX1R antagonist blocked cellular responses in panic and anxiety brain circuits, and imaging data indicated that a selective OX1R antagonist produced a region-dependent inhibition of yohimbine-induced activation in fronto-hippocampal regions as well as in several key components of the extended amygdala^7^. The OX1R inhibition in amygdala is consistent with preclinical studies demonstrating that the orexin system is implicated in amygdala regulation of fear-associated learning^8–10^. In contrast, OX1R antagonists have minimal effect on sleep stages in rodents^11,12^. Overall, there is compelling evidence that overactivation of the OX1R pathway is associated with hyperexcited or hyperactive states; thus, conceptually, a selective OX1R antagonist might normalize overexcited networks without inducing sedation^12^. The extant data also indicated a link between hyperactive orexin system and anxiety- and panic vulnerability in rats and humans^13^. Activation of OX1R was also shown to be a critical component of CO_2_-mediated anxiety and hypertension^16^; notably, CO_2_ inhalation can induce panic-like symptoms in rodents and humans^17^.

JNJ-61393215, a selective OX1R antagonist, is under development for treatment of neuropsychiatric disorders, including stress- and fear-related anxiety syndromes arising within the context of mood and anxiety disorders. Here, we describe the pharmacologic characterization of JNJ-61393215, evaluating in vitro affinity and potency for human and rat OX1R by radioligand-binding and in vitro functional assays. In rats, we measured in vivo target engagement of JNJ-61393215 in the brain and its pharmacodynamic effects on sleep/wake cycles. In our previous studies, we have shown that while selective OX1R antagonism did not affect sleep–wake states, blockade of both OX1R and OX2R elicited a disinhibition of rapid eye movement (REM) sleep at the expense of non-REM (NREM) sleep in rodents^11,12,18^. Therefore, the effect of JNJ-61393215 on sleep was tested in OX2R-knockout (KO) mice to demonstrate in vivo functional target engagement. The activity of JNJ-61393215 was then evaluated on panic-related behaviors induced using the CO_2_ inhalation model.

In addition, we report the first-in-human single-ascending dose (SAD) and multiple-ascending dose (MAD) study wherein the safety, tolerability, pharmacokinetics (PK), and pharmacodynamics (PD) of JNJ-61393215 were evaluated in healthy participants. As inhalation of 35% CO_2_ is a validated model for panic-like symptoms that is also sensitive to the panicolytic effects of benzodiazepines^17,19,20^, we also studied the potential anxiolytic effects of JNJ-61393215, using alprazolam as an active control, in healthy human participants subjected to fear-related anxiogenic effects of CO_2_ inhalation who showed sensitivity to the panic-inducing effects of this challenge during screening.

## Materials and methods

### Animal experiments

All animal experiments were conducted according to the standards recommended by the *Guide for the Care and Use of Laboratory Animals* adopted by the US National Institutes of Health (NIH Publication no. 80-23, revised 1996), and the Janssen Research & Development, LLC, Animal Care and Use Committee approved the protocols. Animals were housed under controlled conditions with a 12-/12-h light/dark schedule and temperature of 22 ± 2 °C. Food and water were provided ad libitum. Experiments were performed after animals had acclimatized for at least 1 week unless stated otherwise.

### Test article

**Fig. 1:**
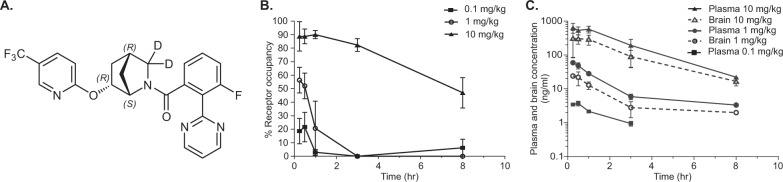
**a** Chemical structure of 3-fluoro-2-(pyrimidin-2-yl)phenyl)((1 S,4 R,6 R)-6-((5-(trifluoromethyl)pyridin-2-yl)oxy)-2-azabicyclo[2.2.1]heptan-2-yl-3,3-d2)methanone). **b** Ex vivo OX1R occupancy with JNJ-61393215 (10, 1, or 0.1 mg/kg, oral administration) in rat tenia tecta: time dependency. **c** Corresponding plasma and brain concentrations (*n* = 3 per time point and dose regimen, brain concentration at 0.1 mg/kg was not measured).

OX1R antagonist JNJ-61393215 (chemical name: 3-fluoro-2-(pyrimidin-2-yl)phenyl)((1 S,4 R,6 R)-6-((5-(trifluoromethyl)pyridin-2-yl)oxy)-2-azabicyclo[2.2.1]heptan-2-yl-3,3-d2)methanone) and its less active enantiomer JNJ-63821238 were synthetized at Janssen Research & Development, LLC (Fig. 1a). Preparation of dosing formulations and reference standards is shown in supplementary text.

### In vitro radioligand-binding assays and in vitro functional assays

These assays were performed as previously described^11^.

### Ex vivo receptor-occupancy assays and plasma/brain exposure measurements

Male Sprague–Dawley rats (300–400 g, Charles River Laboratories, San Diego, CA) received a bolus dose of JNJ-61393215 via a 16-gauge intragastric gavage of 10, 1, or 0.1 mg/kg. Rats were euthanized using CO_2_ and decapitated at defined time points (0.2, 0.5, 1, 3, and 8 h) after administration of test compound (*n* = 3 per time point and dose regimen). Brain and plasma samples were processed as described in supplementary text. Receptor occupancy was measured in the tenia tecta due to the high OX1R-binding density observed in that region, as reported previously^12^.

### Sleep recording and analysis in rats and mice

Sleep experiments were conducted in male Sprague–Dawley rats (350–450 g, Harlan Laboratories, Livermore, CA, USA) and in male C57Bl6 OX2R KO and corresponding wild-type (WT) mice (30–35 g, Charles River Laboratories, San Diego, CA). Sleep was assessed using implanted telemetry to record electroencephalographic (EEG) and electromyographic (EMG) signals using a cross-over design as described previously^21^ and in supplementary text. To determine significant differences between vehicle and compound treatments, a paired Student’s *t* test was performed for NREM and REM latencies and total NREM and REM sleep durations for 6 h. A two-way repeated measure (interaction time × treatment) analysis of variance (ANOVA) followed by a Bonferroni post hoc test was used for NREM and REM sleep duration per 2-h intervals. Differences were determined to be significant if *P* < 0.05. No randomization was used, and blinding was not done.

### 20% CO_2_-induced panic provocation model and cardiovascular response in rats

Experiments were performed in male Sprague–Dawley rats (300–350 g, Harlan Laboratory, Indianapolis, IN) as previously described, using a counterbalanced design^12^. JNJ-63821238, the less active enantiomer of JNJ-61393215, was included as a negative control, and was formulated in the same vehicle. The social interaction test, a fully validated test of experimental anxiety-like behavior in rats, was performed in the open-field arena^22,23^. Both the “experimental” rat and an unfamiliar “partner” rat are placed in the center of the box, and the total duration (seconds) of nonaggressive physical contact (grooming, sniffing, crawling over and under, etc.) initiated by the “experimental” rat is quantified over 5 min. All behaviors were videotaped, and sessions were scored using ANY-maze (Stoelting, Wood Dale, IL) for open field (Stoelting), or by Stephanie D. Fitz (blind to treatments) for social interaction. Each dependent variable for assessment of behavior and radiotelemetry data was respectively analyzed using a one-way ANOVA, or a one-way ANOVA with repeated measures with drug treatment as the main factor and time as repeated measure. In the presence of significant main effects, post hoc tests were conducted using a parametric Fisher’s LSD test. Within-subject’s time effects were also assessed using Dunnett’s one-way analysis with the minute prior to the i.v. infusion used as the control. Statistical significance was accepted with *P* < 0.05. All statistical analyses were carried out using SPSS 13.0 (SPSS Inc., IL, USA), and all graphs were generated using SigmaPlot 2001 (SPSS Inc., IL, USA) or Graphpad Prizm 7 Software Inc. for Windows. No randomization was used.

### Human studies

The study protocols and amendments were reviewed and approved by the Medical Ethics Committee of Stichting Beoordeling Ethiek Biomedisch Onderzoek (Assen, The Netherlands). The studies were conducted in compliance with the Declaration of Helsinki and applicable regulatory requirements. All the human studies were conducted at a single center, Centre for Human Drug Research, Leiden, the Netherlands. Written informed consent was obtained from all participants prior to study enrollment. The study design is summarized in Supplementary Fig. S1.

### Single-ascending dose (SAD) study of oral JNJ-61393215 in healthy human volunteers

The SAD study was a phase-1, randomized, double-blind, placebo-controlled study to assess safety, tolerability, and PK of single increasing oral doses of JNJ-61393215 in healthy adult participants (NCT02812251). This was a 3-part study with a total enrollment of 80 participants. The number of participants in each part is the customary sample size employed in such studies. Randomization was used in the assignment of participants to treatment groups using a computer-generated randomization schedule. In Part 1, the single-dose escalation consisted of eight cohorts of healthy male volunteers. In each cohort, six participants received JNJ-61393215 and two participants received matching placebo under fasted conditions. JNJ-61393215 (1, 2, 6, 15, 30, 45, 60, and 90 mg) or placebo was administered as a single oral dose. The NeuroCart test battery^24^ was applied to characterize the PD profile of JNJ-61393215. CSF levels of JNJ-61393215 and food effect were assessed in Parts 2 and 3, respectively, and those results will be described in separate publications. The study was conducted in a double-blind manner for parts 1 and 3. Blinding was not used in part 3 because all participants received the same dose.

### Multiple-ascending dose (MAD) study of oral JNJ-61393215 in adults

The MAD study was a randomized, double-blind, placebo-controlled study to investigate safety and tolerability, PK, and PD of JNJ-61393215 in healthy participants under fasted conditions (NCT03007693). Part 1 of the MAD study included four cohorts of male participants (eight participants/cohort, six active, two placebo) with dose levels of 5 mg (Cohort 1), 15 mg (Cohort 2), 45 mg (Cohort 3), and 90 mg (Cohort 4). The number of participants per cohort is the customary sample size employed in early-development dose-escalation studies. Randomization was used in the assignment of participants to treatment groups using a computer-generated randomization schedule. The study was conducted in a double-blind manner.

### 35% double-inhalation CO_2_ panic provocation model in humans

Part 2 (cohort 1) was conducted to establish the anxiolytic PD properties of JNJ-61393215 using the CO_2_ Tolerance Tester (CTT), a research instrument developed by Maastricht Instruments in collaboration with Maastricht University to induce panic attacks by the protocolized administration of inhaled 35% CO_2_. The CO_2_ challenge procedure was similar to that described in Leibold et al.^17^.

Healthy male participants sensitive to the anxiogenic effects of 35% CO_2_ inhalation at screening were randomized to receive JNJ-61393215 (25 mg or 90 mg of QD: extrapolated receptor occupancy at peak concentrations of 93% and 98.5%, respectively), alprazolam (1 mg of bid), or placebo for 7 days. Sensitivity to the CO_2_ challenge at screening was defined as a change in Panic Symptom List (PSL)-IV score ≥4 with ≥1-point increase for at least four symptoms, and an increase of at least 25 mm on the Visual Analogue Scale (VAS) for fear-related symptoms^17,24^.

Part 2 of the study applied a four-treatment 3-arm 2 × 2 cross-over design, and each participant was randomized to receive either placebo or one of the three active treatments (Supplementary Fig. S1). Assuming that the mean (SD) of paired difference between JNJ groups and placebo observed for this study is −7(10.3), 12 participants achieve 84% power using a paired *t* test at a one-sided alpha of 0.10. A sample size of 36 was used for a four-treatment 3-arm 2 × 2 cross-over design.

Participants underwent a 35% CO_2_ double-inhalation challenge using the CTT methodology after 6 days of dosing with placebo or active treatment in each cross-over period, and the fear symptoms induced by the CO_2_ challenge were measured immediately after CO_2_ inhalation using the panic symptom list IV (PSL-IV). The CO_2_ challenge was performed 2.5 h after dosing with JNJ-61393215; alprazolam was used as active comparator to establish assay sensitivity as prior studies had shown fear-reducing properties of alprazolam in the CO_2_ challenge after a single dose. Within each arm, the active treatment was compared with matched placebo on PSL-IV total score by using a linear mixed-effect model, controlling for treatment, period, and sequence as a fixed effect, participant as a random effect, and baseline score as a covariate (see supplementary text for eligibility criteria and PK and PD assessments).

## Results

### Preclinical studies

#### In vitro affinity binding and potency of JNJ-61393215 to OX1R

JNJ-61393215 demonstrated high-affinity binding to the human OX1R and rat OX1R (rOX1R), with negative log of inhibition constant (pK_i_) values of 8.17 ± 0.09 and 8.13 ± 0.09, respectively. The binding selectivity of JNJ-61393215 for human OX1R compared with human OX2R was substantial (pK_i_ values of 8.17 vs. 6.12). The negative log of the functional equilibrium dissociation constant (pK_B_) values for JNJ-61393215 showed good correlation to its pK_i_ values for human and rOX1R (pK_B_: OX1R, 7.76 ± 0.05; rOX1R, 7.72 ± 0.12). The binding selectivity of JNJ-61393215 at OX1R compared with OX2R was confirmed at the functional level (OX1R pK_B_ = 7.76 vs. OX2R pK_B_ = 6.01).

JNJ-61393215 was screened in a panel of other receptors, ion channels, transporters, and kinases (Eurofins, France), including adenosine (A_1_, A_2A_, and A_3_), adrenergic (α_1_, α_2_, and α_1_), angiotensin (AT_1_), dopamine (D_1_ and D_2_), bradykinin (B_2_), cholecystokinin (CCK_A_), galanin (GAL_2_), melatonin (ML_1_), muscarinic (M_1_, M_2_, and M_3_), neurotensin (NT_1_), neurokinin (NK_2_ and NK_3_), opiate (μ, κ, and δ), serotonin (5-HT_1A_, 5-HT_1B_, 5-HT_2A_, 5-HT_3_, 5-HT_5A_, 5-HT_6_, and 5-HT_7_), somatostatin, vasopressin (V_1a_), norepinephrine transporter, dopamine transporter, and ion channels (sodium, calcium, potassium, and chloride), and had no significant affinity for any of those binding sites tested (<50% inhibition at 10 μM) other than for OX1R.

#### Rat brain OX1R occupancy and plasma and brain exposure of JNJ-61393215

Oral administration of JNJ-61393215 resulted in time- and dose-dependent occupancy of the OX1R in rat tenia tecta, indicating sufficient oral bioavailability and brain penetration (Fig. 1b). Following acute oral administration of 10 mg/kg, maximal OX1R occupancy was observed at 15 min (89% ± 6%, Fig. 1b). The level of OX1R occupancy remained above 47% for the first 8 h, and then dropped below the measurable level of occupancy at 16 h. The brain-to-plasma-concentration ratio ranged from ~0.4 to 0.7 (Fig. 1c). Overall, the general trend between receptor-occupancy level and plasma exposure was similar (Fig. 1c). The plasma PK was linear, and the plasma concentration associated with 50% occupancy (EC_50_) was 34 ng/mL (95% confidence intervals, 21–56 ng/mL).

#### Effect of JNJ-61393215 on sleep parameters in rats

Administration of JNJ-61393215 (10 mg/kg) at the onset of the dark phase showed no alteration in either NREM or REM sleep duration in the 6-h recording period, and there was a significant decrease in the latency of both NREM (*P* < 0.01) and REM sleep (*P* < 0.05; Supplementary Table S1).

#### JNJ-61393215 selectively promotes REM sleep in OX2R-knockout (KO) mice

Functional target engagement in vivo was investigated in a model of permanent inhibition of OX2R, and the effects of JNJ-61393215 on sleep–wake states were examined in KO mice lacking the OX2R relative to their corresponding WT mice. In OX2R KO mice, oral dosing of JNJ-61393215 (30 mg/kg) compared with vehicle at 2 h into the light phase significantly reduced the latency for REM sleep (*P* < 0.01) and prolonged the time spent in REM sleep during the first 2 h post treatment (*P* < 0.05; Supplementary Fig. S2A), but did not alter REM sleep in WT mice (*P* > 0.05; Supplementary Fig. 2B). As opposed to REM sleep parameters, NREM sleep latency and duration were not significantly affected by the JNJ-61393215 in either genotype (*P* > 0.05; Supplementary Figs. S2C and S2D).

#### Effect of JNJ-61393215 on CO_2_-induced fear and cardiovascular response in rats

**Fig. 2:**
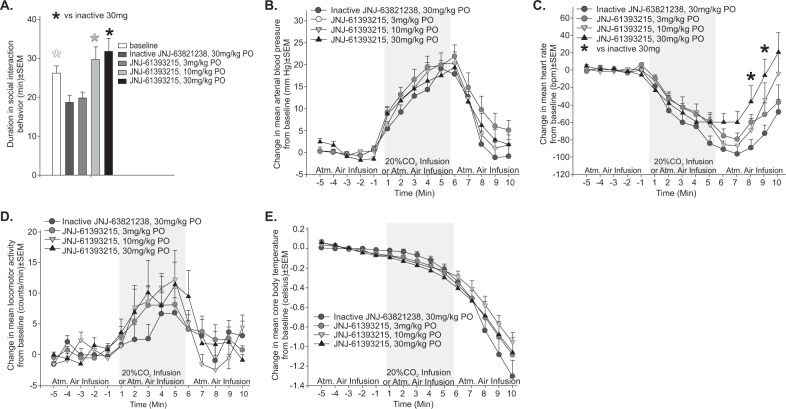
Effect of JNJ-61393215 (3, 10, and 30 mg/kg, oral administration) and its less active enantiomer JNJ-63821238 (30 mg/kg, oral administration) on CO_2_-induced panic responses. Line and bar graphs in all panels represent (**a**) social interaction time (min), (**b**) mean arterial blood pressure (mm Hg), **c** heart rate (beats per min), **d** general locomotor activity (counts/min), and **e** core body temperature (celsius) of rats pretreated with 30 mg/kg of JNJ-63821238, the less active enantiomer of JNJ-61393215 (control group, *n* = 11), or JNJ-61393215 compound at 3 doses (3, 10, and 30 mg/kg with *n* = 10/group, one rat was discontinued during crossover due to technical complications) p.o. 30 min prior to onset of 10 min of atmospheric air infusions, followed by a 5-min 20% CO_2_ (normoxic) air infusion (gray shaded region), then another 5-min infusion of atmospheric air. Physiology was obtained from radiotelemetry probes with a pressure transducer in the femoral artery for cardiovascular measures and an internal thermistor for core body temperature. *P < 0.05 and see “Results” for detailed statistical analysis.

JNJ-61393215 was tested in a rat model of 20% CO_2_-induced panic, using oral doses of 3, 10, and 30 mg/kg administered 30–50 min prior to the CO_2_ challenge (when a baseline was established for physiological measures, see Fig. 2). The less active enantiomer of JNJ-61393215, JNJ-63821238, was included as a negative control.

Repeated measure ANOVA with treatment as the main factor and time as the repeated measure revealed the following:

- A one-way ANOVA revealed differences between groups F(4,51) = 5.2, *P* < 0.001 (Fig. 2a). Ficher’s LSD post hoc test revealed that the CO_2_-induced reduction in social interaction behaviors (indicating fear state) was blocked in rats pretreated with the 10 or 30 mg/kg dose of JNJ-61393215.
- A significant increase in mean arterial blood pressure over time F(14,518) = 83.1, *P* < 0.001, but no treatment × time interaction was observed F(42,518) = 0.7, *P* = 0.918. There were also no significant differences in mean arterial blood pressure noted at baseline between treatment groups F(3,40) = 0.5, *P* = 0.708 (Fig. 2b).
- A significant decrease in heart rate over time F(14,518) = 53.6, *P* < 0.001, and a treatment × time interaction was observed F(42,518) = 2.1, *P* < 0.001, and a Fisher’s least-square difference (LSD) post hoc test protected by an ANOVA at each time point revealed that JNJ-61393215 attenuated CO_2_-induced bradycardia responses at two time points (8 and 9 min) post CO_2_ (*P* < 0.05, Fig. 2c). Also, no differences in heart rate were detected at baseline between treatment groups F(3,40) = 0.5, *P* = 0.667.
- A significant increase was detected in general motor activity over time F(14,518) = 10.7, *P* < 0.001, but no treatment × time interaction was observed F(42,518) = 0.9, *P* = 0.658. No differences in general motor activity were detected at baseline between treatment groups F(3,40) = 0.4, *P* = 0.757 (Fig. 2d).
- A significant decrease was detected in core body temperature over time F(14,518) = 146.3, *P* < 0.001, but no treatment × time interaction was observed F(42,518) = 1.2, *P* = 0.202. Also, no differences in core body temperature were detected at baseline between treatment groups F(3,40) = 0.2, *P* = 0.912 (Fig. 2e).

Mean total and unbound JNJ-61393215 plasma levels measured at 30 min at 10 mg/kg were 535 ng/mL and 86 ng/mL, respectively, while at 30 mg/kg, mean total and unbound levels were 2698 ng/mL and 432 ng/mL, respectively (Supplementary Table S2).

### Clinical studies

#### Pharmacokinetic profiles and safety of single-ascending dose (SAD) of JNJ-61393215 in humans (part 1)

In the SAD study, 72 male participants (aged 18–52 years, mean [SD] age: 31 [15.06] years; BMI: 23.1 kg/m^2^, median 22.7; range 19–30) were included. Females were excluded due to lack of preclinical reproductive and developmental toxicity results available at the time the study was conducted. The study consisted of 8 cohorts; 6 male participants per cohort received a single dose of JNJ-61393215, ranging from 1 to 90 mg (*n* = 48). The majority (87.5%) of participants in the JNJ-61393215 treatment groups were Caucasians. The mean values of *C*_max_, AUC_last_, and AUC_∞_ of JNJ-61393215 increased dose-proportionally with increasing dose up to 30 mg (Supplementary Table S3).

The most frequently reported treatment-emergent adverse events (TEAEs) in participants dosed with JNJ-61393215 (1–90 mg) were somnolence (*n* = 8, 16.7%) and headache (*n* = 23, 47.9%). In the placebo group, six (33%) participants experienced headache, and three (16.7%) participants reported somnolence (Supplementary Table S4).

#### Pharmacokinetic profiles and safety of multiple-ascending dose (MAD) of JNJ-61393215 in humans (parts 1 and 2)

**Fig. 3:**
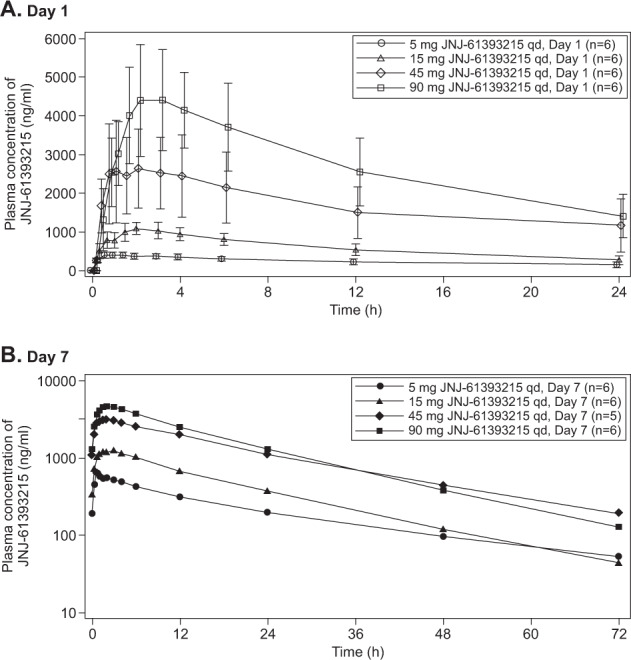
Mean plasma-concentration–time profiles of JNJ-61393215 on day 1 (**a**, linear [SD]) and day 7 (**b**, semilogarithmic) following multiple oral doses of 5, 15, 45, and 90 mg of JNJ-61393215 QD in healthy male participants (Part 1).

Overall, 32 male participants (mean [SD] age: 30.5 [11.2] years) in Part 1 and 39 male participants (mean [SD] age: 28.2 [8.0] years) in Part 2 were included in the MAD study. In the JNJ-61393215 treatment groups, the majority (~80%) of participants were Caucasians with mean BMI of 22.9 (2.8) kg/m^2^ (range 17.9–29.4). On days 1 and 7, mean *C*_max_ and AUC_24h_ increased with increasing doses (Fig. 3; Supplementary Table S5). Overall, the mean values for the dose-normalized PK parameters on days 1 and 7 slightly decreased with increasing doses, suggesting a less-than dose-proportional increase over the dose range of 5–90 mg of JNJ-61393215 (Supplementary Table S5).

For Part 1, the most common TEAEs (reported by ≥10% of participants) in all JNJ-61393215 treatment groups were headache (33.3%), somnolence (29.2%), nausea (16.7%), nasopharyngitis (16.7%), dysgeusia (12.5%), and epistaxis (8.3%; Supplementary Table S4). In the placebo group, three participants (37.5%) experienced headache and three participants experienced somnolence (37.5%) and two (25%) participants experienced dysgeusia (Supplementary Table S4). None of the TEAEs were considered severe. There were no consistent treatment-related effects in clinical laboratory parameters, and no abnormalities were observed during physical examination.

#### Human pharmacodynamic results (MAD study part 2)

**Fig. 4:**
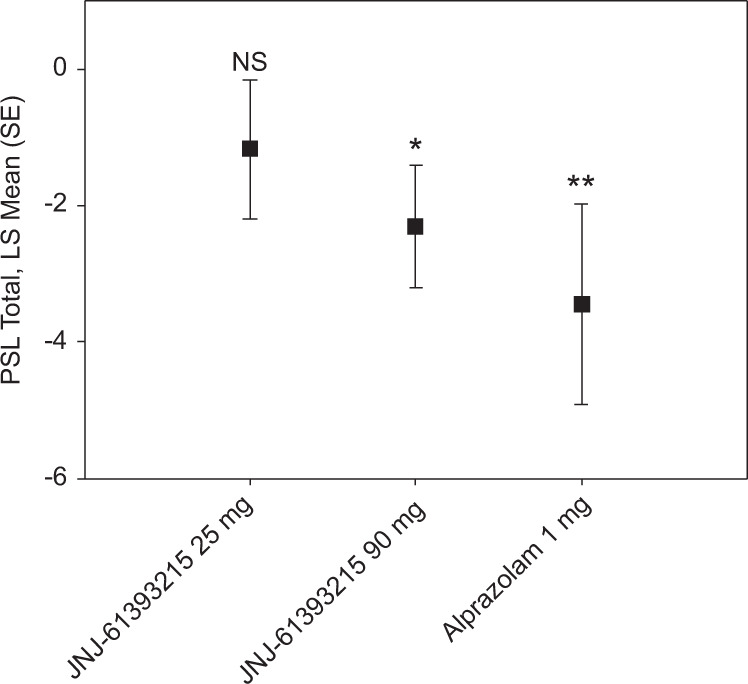
Changes in subjective fear as reflected by the panic symptom list IV (PSL-IV) total scores after the CO_2_ challenge and JNJ-61393215 (25 or 90 mg of QD) or alprazolam (1 mg of BID). LS mean difference between each active treatment and placebo; *P < 0.02, **P < 0.03, NS not significant.

Treatment with JNJ-61393215 (90 mg) significantly reduced fear symptoms induced by double inhalation of 35% CO_2_ in healthy volunteers according to the primary outcome measure, namely the change PSL-IV total score (difference of least-square [LS] means (SE): −2.3 (0.9), *P* < 0.02); a significant anxiolytic effect was also demonstrated for a therapeutic dose of active comparator alprazolam (difference of LS means (SE): −3.4 (1.5), *P* < 0.03), demonstrating assay sensitivity. Individual item changes after active treatment or placebo are presented in Table 1 in a descriptive manner, to highlight the clinical implication of the PSL-IV total score analysis. Statistical analyses were not performed on individual item change, as the study was not powered to look at individual item changes after applying appropriate corrections for multiple comparisons given the relatively small sample size (which was based on power calculations using the estimated PSL-IV total score change only). The anxiolytic effect of JNJ-61393215 was observed in most participants, and was driven by a reduction in severity of 9/13 items of the PSL-IV, suggesting a broad anxiolytic effect (Table 1). The low dose of JNJ-61393215 (25 mg) was associated with a nonsignificant decrease in fear symptoms (Fig. 4). Mean unbound *C*_max_ 90 mg (68 ng/mL) on day 7 was comparable to the mean unbound *C*_max_ obtained in the CO_2_-induced reduction in social interaction behavior paradigm at 10 mg/kg (86 ng/mL) in rats (Supplementary Table S2).

**Table 1:** PSL-IV individual item change scores post CO_2_ challenge.

	Placebo	JNJ-61393215 25 mg of QD	Placebo	JNJ-61393215 90 mg of QD	Placebo	Alprazolam 1 mg of BID
Dizziness	1.9 (1.08)	1.3 (1.22)	2.3 (1.06)	1.8 (1.09)	2.1 (0.92)	1.7 (1.19)
Choking/gasping for breath	1.8 (0.83)	1.9 (0.90)	2.5 (1.38)	1.8 (1.28)	2.1 (0.92)	1.9 (1.30)
Hot flashes/cold shivers	0.3 (0.75)	0.3 (0.65)	0.9 (1.56)	0.5 (1.33)	0.8 (1.25)	0.2 (0.40)
Nausea	0.4 (0.51)	0.2 (0.39)	0.7 (1.23)	0.5 (1.33)	0.3 (0.61)	0.1 (0.30)
Palpitations	1.9 (1.08)	1.3 (1.15)	1.8 (1.27)	1.8 (1.28)	1.9 (0.95)	1.3 (0.90)
Sweating	0.7 (0.49)	0.8 (0.94)	1.3 (1.29)	0.9 (0.86)	1.3 (1.14)	1.0 (0.89)
Shortness of breath	1.6 (1.00)	1.8 (0.97)	2.3 (1.15)	2.1 (1.32)	2.2 (0.89)	1.9 (1.22)
Numb/tingling	0.8 (0.94)	0.7 (1.07)	1.5 (1.0)	1.2 (1.36)	0.9 (0.95)	0.7 (0.79)
Depersonalization/derealization	0.5 (0.80)	0.6 (0.79)	1.2 (1.47)	1.2 (1.41)	1.1 (1.10)	0.8 (0.98)
Fear of dying	0.1 (0.29)	0.1 (0.29)	0.3 (0.89)	0.4 (1.12)	0.3 (0.73)	0.1 (0.30)
Fear of losing control	0.3 (0.65)	0.3 (0.65)	0.4 (0.90)	0.4 (0.87)	0.3 (0.61)	0.4 (0.50)
Chest pain discomfort	0.3 (0.45)	0.2 (0.39)	0.8 (1.14)	0.7 (1.18)	0.1 (0.36)	0 (0)
Trembling/shaking	1.3 (0.87)	1.3 (0.97)	1.6 (1.51)	1.4 (1.39)	1.1 (1.27)	1.2 (1.54)

*BID* twice daily, *PSL-IV* panic symptom list IV, *SD* standard deviation, *QD* once daily.

Data are represented as mean (SD).

#### Cardiovascular parameters

JNJ-61393215 and alprazolam did not have any significant effects on cardiovascular parameters, such as blood pressure or heart rate (data not shown). Considerable variability of physiological parameters at baseline and after the CO_2_ challenge was observed across the study population, which is consistent with the results of a recent meta-analysis^25^.

## Discussion

In the preclinical studies described in this report, we characterized the novel selective OX1R antagonist, JNJ-61393215, as a clinical candidate for the treatment of panic and anxiety disorders. We also demonstrated that pretreatment of rats with JNJ-61393215 attenuates the anxiety- and panic-like responses elicited by 20% CO_2_ inhalation. Safety, PK, and PD properties of JNJ-61393215 in first-in-human single- and multiple-ascending dose studies were described, and an anxiolytic effect of JNJ-61393215 was demonstrated in healthy participants using a modified CO_2_ challenge assay to induce panic symptoms. This is the first report of anxiolytic effects of a selective OX1R antagonist in humans.

In the in vitro studies, JNJ-61393215 demonstrated high affinity, potency, and selectivity for OX1R with an approximate 2-log selectivity ratio relative to OX2R. The in vivo occupancy of OX1R as assessed by ex vivo receptor analysis showed time- and dose-dependent OX1R occupancy in rat brain. In the rat study, JNJ-61393215 showed no effect on spontaneous sleep; however, a decrease in latency to NREM and REM was observed and was similar to previously observed reports with a different OX1R antagonist JNJ-54717793^11^. Our preclinical findings corroborate the results of other studies wherein selective OX1R antagonists, in contrast to selective OX2R antagonists, minimally affect sleep–wake states in baseline conditions^21,26,27^. EEG was obtained during the rat’s dark/active phase that also corresponds to the daytime/active phase in humans when the drug was given. The dark phase in rodents is the optimal phase to detect any hypnotic activity for orexin-receptor antagonists (due to higher orexin levels) and the only circadian phase where all orexin KO (peptide and receptors) show the sleep phenotype. We also tested JNJ-61393215 in WT mice at a higher dose during the light phase, in which it proved to be devoid of sleep-promoting effects. Consistent with our previous results on simultaneous pharmacological OX1R and OX2R blockade, JNJ-61393215 promoted REM sleep in OX2R KO mice, but not in WT mice, demonstrating that the compound engaged OX1Rs and elicited a specific OX1R blockade.

While JNJ-61393215 showed no effect on baseline cardiovascular, temperature, or locomotor activity, it blocked CO_2_-induced fear in the social interaction test at 10 mg/kg and 30 mg/kg. In addition, an attenuation of the bradycardia response to CO_2_ challenge was also observed at 30 mg/kg dosing.

The efficacy observed in the rat CO_2_ challenge is unlikely to be driven by nonspecific effects, as demonstrated by the lack of effect on baseline locomotor or autonomic activity, and by the lack of effect of the less active enantiomer JNJ-63821238. A high level of target engagement (>90%) was required to demonstrate efficacy in the CO_2_ challenge, as previously observed with other OX1R antagonists (SB-334867, Compound 56 and JNJ-54717793)^11,12,28^. These findings are consistent with the hypothesized role of OX1R signaling in the mechanism underlying CO_2_ challenge-induced fear, as exposing rats to higher concentration of CO_2_ depolarizes orexin neurons by interacting with pH/CO_2_-chemosensitive K^+^ channels^29^. This results in subsequent release of orexin at postsynaptic sites in brain and brainstem regions to mobilize anxiety-like behavior, hypertension, and increased ventilatory responses^28,30^.

Notably, the unbound mean JNJ-61393215 plasma concentrations at which CO_2_-induced fear in rats was inhibited (86–432 ng/mL), were comparable to those associated with the reduction of experimental 35% CO_2_-induced fear in humans (68 ng/mL). In fact, the unbound plasma exposure in rats predicted the exposure at which inhibition of CO_2_-induced fear could be expected in humans.

In humans, JNJ-61393215 demonstrated an acceptable safety profile for both after single-dose and multiple-dose administration up to 90 mg, as suggested by the findings in the SAD and the MAD studies in humans. The generalizability of these results is limited; however, since the SAD and MAD studies did not include women, the preclinical reproductive and developmental toxicology experiments had not been completed when those studies were executed. It is important to note that the maximal doses administered in the SAD and the MAD studies, which correspond to extrapolated receptor occupancy >95%, were not associated with somnolence, providing evidence in humans that the soporific properties of dual orexin-receptor antagonists are associated with orexin-2 receptors. The majority of TEAEs were mild in severity, and their frequency was comparable to placebo. After administration of a single dose of JNJ-61393215, peak and total exposure increased in a dose-proportional manner up to 30 mg. JNJ-61393215 did not produce any pharmacodynamic effects as a single dose up to 90 mg in healthy male subjects, consistent with preclinical data that show no detectable pharmacodynamic or behavioral effects in naive/unchallenged animals.

The safety profile of JNJ-61393215 is consistent with the recently reported clinical data for another selective OX1R antagonist, ACT-539313, where the most commonly reported adverse events were somnolence and headache^31^.

JNJ-61393215 also showed anxiolytic properties in a human experimental model of panic induced by inhalation of high concentrations of CO_2_. Several aspects related to the CO_2_ challenge merit comment, especially in light of the methodological heterogeneity of prior studies that implemented similar paradigms. In the human MAD study, we opted for double-breath inhalation of 35% CO_2_, which is considered an experimental model of panic^17^, in contrast to other CO_2_-challenge studies that used 5% or 7% CO_2_ inhaled over prolonged periods, and has been proposed as a model for generalized anxiety disorder^32^. CO_2_ is hypothesized to acutely disturb the brain acid–base homeostasis in both medullary respiratory neurons and serotonergic midbrain raphe neurons.

The choice of a challenge model more relevant for panic symptoms to test PD effects of JNJ-6139215 is consistent with the mechanism of action of OXR1 inhibition as well as prior preclinical studies^21^. The 35% double-inhalation CO_2_ challenge has been extensively validated with several anxiolytic treatments, such as acute dosing of benzodiazepines, chronic dosing with selective serotonin-reuptake inhibitors, and chronic dosing with tricyclic antidepressants^17,19,20^. The panicolytic effects of JNJ-61393215 and alprazolam were also tested at steady state and not after a single dose, while previous studies with benzodiazepines have tested their panicolytic effects in the CO_2_ challenge only after a single dose. Together, this study provided further validation for the CO_2_ challenge as a test sensitive to the effects of alprazolam even after multiple doses, supporting the translational value of this challenge in anxiolytic drug development.

The incomplete cross-over design was an additional strength to the conduct of CO_2_-challenge experiments, and allowed testing of each experimental treatment versus placebo in a relatively small number of participants. Also, we limited enrollment to participants who were sensitive to CO_2_ inhalation at screening in the MAD study. Previous studies have shown that healthy volunteers manifest lower sensitivity to the panic-like effects of CO_2_ inhalation than patients with panic disorder^33^; therefore, the implementation of an enrichment strategy was deemed necessary to avoid potential floor effects, which may have compromised the detection of potential PD anxiolytic effects induced by administration of JNJ-61393215 and alprazolam. JNJ-61393215 at 90 mg showed a robust anxiolytic effect, driven by a decrease in most items on the PSL-IV, similar to the active comparator, alprazolam, supporting the potential anxiolytic effect properties of OX1R antagonists. The reduction in anxiety symptoms observed after administration of JNJ-61393215 90 mg and alprazolam was not paralleled by any changes in cardiovascular parameters: we observed marked interindividual variability in the changes in blood pressure and heart rate elicited by CO_2_ inhalation, as well as in the effects of JNJ-61393215 and alprazolam on those measures, a finding consistent with a recent meta-analysis that highlighted the large variability and limited interpretability of the effects of the CO_2_ challenge on cardiovascular parameters^25^.

Notably, the extant preclinical data suggest that OX1R antagonists may hold anxiolytic effects that extend beyond panic anxiety. For example, in rodents, the autonomic and behavioral responses to stress were attenuated by pretreatment with OX1R antagonists^8^.

In conclusion, we demonstrated for the first time in humans the panicolytic effects of a selective OX1R antagonist, JNJ-61393215, with an acceptable safety profile. In addition, this study supported the validity of the CO_2_-exposure challenge as a translational, cross-species experimental model for panic. These results collectively support further evaluation of JNJ-61393215 efficacy in clinical studies as a potential treatment for patients with panic or other anxiety disorders, and in mood disorders associated with anxious hyperarousal.

## Supplementary information

Supplementary Information
