# Supplementary material for: Translational evaluation of novel selective orexin-1 receptor antagonist JNJ-61393215 in an experimental model for panic in rodents and humans
Source: Transl Psychiatry. 2020 Sep 7;10:308. doi: 10.1038/s41398-020-00937-9 (PMC7477545; doi:10.1038/s41398-020-00937-9)
Supplement: Supplementary file 1 — Supplementary Information [file 41398_2020_937_MOESM1_ESM.docx]

**Supplementary Tables**

#### **Supplementary Table** **S1. Effects of the OX1R antagonist JNJ-61393215 (10 mg/kg oral administration) on sleep parameters in rats (dark phase)**

|  | **Vehicle** | **JNJ-61393215** |
| --- | --- | --- |
| **NREM Latency (min)** | 33.0 ± 2.5 | 22.6 ± 3.2** |
| **NREM Duration (min)** |  |  |
| 1-2 h | 27.0 ± 2.4 | 26.7 ± 2.3 |
| 3-4 h | 38.0 ± 1.8 | 37.1 ± 2.7 |
| 5-6 h | 35.5 ± 3.1 | 42.2 ± 3.1 |
| Total 6 h | 100.5 ± 2.7 | 106.0 ± 4.1 |
| **REM Latency (min)** | 54.4 ± 7.6 | 39.3 ± 6.9* |
| **REM Duration (min)** |  |  |
| 1-2 h | 4.5 ± 1.0 | 6.5 ± 0.7 |
| 3-4 h | 8.2 ± 1.4 | 8.8 ± 1.2 |
| 5-6 h | 6.3 ± 1.3 | 9.4 ± 1.3 |
| Total 6 h | 19.0 ± 1.8 | 24.7 ± 2.1 |

Rats were orally dosed with JNJ-61393215 (10 mg/kg) or vehicle at the onset of the dark phase. Data represented as means ± SEM. of 8 animals and are expressed in min. *p<0.05 and **p<0.01 vs vehicle as determined by paired Student’s t test.

*NREM* non-rapid eye movement; *OX1R* orexin-1 receptor; *REM* rapid eye movement; *SEM* standard error mean

#### **Supplementary Table S2. Total and unbound JNJ-61393215 plasma concentrations in rat (30 min) and human (C_max_)**

| **Species** | **JNJ-61393215 dose** | **Mean ng/mL** | |
| --- | --- | --- | --- |
|  |  | **Total** | **Unbound** |
| Rat | 10 mg/kg at 30 min | 535 | 86 |
|  | 30 mg/kg at 30 min | 2698 | 432 |
| Human | SAD (90 mg) C_max_ | 4575 | 92 |
|  | MAD (90 mg) day 7 C_max_ | 3423 | 68 |

C_max_, maximum plasma concentration; SAD, single-ascending dose; MAD, multiple-ascending dose

#### **Supplementary Table S3. Pharmacokinetic results of JNJ-61393215 following single oral administration under fasted conditions**

| **SAD, JNJ-61393215** | | | | | | | | |
| --- | --- | --- | --- | --- | --- | --- | --- | --- |
| **PK parameters** | **Part 1 (fasted)** | | | | | | | |
|  | **Cohort 1**  **1 mg** | **Cohort 2**  **2 mg** | **Cohort 3**  **6 mg** | **Cohort 4**  **15 mg** | **Cohort 5**  **30 mg** | **Cohort 6**  **45 mg** | **Cohort 7**  **60 mg** | **Cohort 8**  **90 mg** |
| **N** | 6 | 6 | 6 | 6^a^ | 6 | 6 | 6 | 6 |
| **C_max_, ng/mL** | 97.4 ± 10.1 | 177 ± 24.3 | 643 ± 111 | 1390 ± 171 | 2850 ± 701 | 3122 ± 336 | 3552 ± 736 | 4497 ± 664 |
| **t_max_, h,**  **median (range)** | 1.00 (1.00 – 2.00) | 1.26 (1.00 – 2.48) | 1.00 (1.00 – 1.02) | 1.50 (0.50 – 3.03) | 1.50 (1.00 – 6.00) | 2.26 (1.00 – 3.10) | 2.25 (1.00 – 6.00) | 2.25 (1.03 – 6.00) |
| **C_last_, ng/mL** | 5.56 ± 4.13 | 9.99 ± 6.60 | 28.9 ± 38.8 | 155 ± 137 | 281 ± 187 | 103 ± 128 | 148 ± 165 | 104 ± 66.1 |
| **AUC_last_, ng.h/mL** | 1974 ± 484 | 3795 ± 1129 | 11804 ± 4560 | 36101 ± 8898 | 72100 ± 23112 | 55232 ± 20288 | 70482 ± 30326 | 78253 ± 18009 |
| **AUC_∞_, ng.h/mL** | 2148 ± 636 | 4068 ± 1323 | 12778 ± 6127 | 35858 ± 5760 | 82306 ± 30685 | 58186 ± 24756 | 74467 ± 35958 | 80461 ± 19572 |
| **t_1/2term_, h** | 18.2 ± 5.7 | 16.7 ± 4.4 | 16.0 ± 6.6 | 24.6 ± 12.4 | 21.5 ± 6.7 | 14.5 ± 5.3 | 15.0 ± 4.2 | 13.6 ± 2.3 |
| **CL/F, L/h** | 0.501 ± 0.144 | 0.545 ± 0.211 | 0.551 ± 0.221 | 0.427 ± 0.0685 | 0.419 ± 0.180 | 0.874 ± 0.295 | 0.922 ± 0.306 | 1.18 ± 0.309 |
| **Vd/F, L** | 12.2 ± 1.26 | 12.0 ± 1.56 | 11.3 ± 1.91 | 11.9 ± 1.30 | 11.6 ± 1.30 | 16.6 ± 2.34 | 18.5 ± 3.32 | 22.3 ± 2.21 |
| **CLcr, mL/min** | 142 ± 22.8 | 147 ± 39.6 | 142 ± 17.5 | 136 ± 30.2 | 121 ± 44.5 | 117 ± 11.0 | 132 ± 22.0 | 136 ± 31.2 |
| **C_max_ _unbound, dose normalized,_ ng/mL** | 1.40 ± 0.360 | 1.50 ± 0.308 | 2.10 ± 0.217 | 1.47 ± 0.187 | 1.89 ± 0.562 | 1.81 ± 0.334 | 1.45 ± 0.469 | 1.52 ± 0.334 |
| **AUC_last, unbound, dose normalized,_  ng.h/mL/mg** | 27.1 ± 2.73 | 31.0 ± 4.61 | 37.4 ± 8.41 | 37.8 ± 6.82 | 45.7 ± 8.90 | 31.0 ± 8.14 | 28.2 ± 10.9 | 25.8 ± 4.74 |

^a^ N=5 for AUC_∞_, CL/F, Vd/F and AUC_∞_

AUC_∞_, area under the plasma concentration-time curve from time 0 to infinite time; AUC_last_, area under the plasma concentration-time curve from time 0 to time of the last quantifiable concentrations; C_last_, last quantifiable plasma concentration; C_max_, maximum plasma concentration; CLcr, creatinine clearance; CL/F, total clearance of drug after extravascular administration; SAD, single-ascending dose; t_1/2term_**,** elimination half-life associated with the terminal slope (λz) of the semi-logarithmic drug concentration-time curve; t_max_, time to reach maximum plasma concentration; Vd/F, apparent volume of distribution

**Supplementary Table S4. Summary of treatment-emergent adverse events after single or multiple-administration of JNJ‑61393215 doses by preferred term (in ≥5% participants)**

| **SAD** | | | | | | | | | | | | | | |
| --- | --- | --- | --- | --- | --- | --- | --- | --- | --- | --- | --- | --- | --- | --- |
|  | **Placebo** | **JNJ-61393215** | | | | | | | | | | | | |
|  |  | **1 mg** | | **2 mg** | | **6 mg** | | **15 mg** | **30 mg** | | **45 mg** | **60 mg** | **90 mg** | **Total** |
| **Total**  Any TEAE, n (%) | **18**  11 (61) | **6**  1 (16.7) | | **6**  2 (33.3) | | **6**  3 (50) | | **6**  1 (16.7) | **6**  6 (100) | | **6**  3 (50) | **6**  3 (50) | **6**  4 (66.7) | **62**  34 (54.8) |
| Headache | 6 (33.3) | 0 | | 0 | | 0 | | 0 | 2 (33.3) | | 1 (16.7) | 0 | 0 | 8 (12.9) |
| Somnolence | 3 (16.7) | 0 | | 0 | | 2 (33.3) | | 1 (16.7) | 0 | | 2 (33.3) | 2 (33.3) | 1 (16.7) | 9 (14.5) |
| **MAD, Part 1 study** | | | | | | | | | | | | | | |
|  | **Placebo** | | **JNJ-61393215** | | | | | | | | | **Active Total** | | **Total** |
|  |  | | **5 mg** | | **15 mg** | | **45 mg** | | | **90 mg** | |  | |  |
| **Total** | **8** | | **6** | | **6** | | **6** | | | **6** | | **24** | | **32** |
| Any TEAE,  n (%) | 6 (75) | | 6 (100) | | 3 (50) | | 6 (100) | | | 5 (83.3) | | 20 (83.3) | | 26 (81.3) |
| Headache | 3 (37.5) | | 2 (33.3) | | 1 (16.7) | | 2 (33.3) | | | 3 (50) | | 8 (33.3) | | 11 (34.4) |
| Somnolence | 3 (37.5) | | 1 (16.7) | | 1 (16.7) | | 3 (50) | | | 2 (33.3) | | 7 (29.2) | | 10 (31.3) |
| Dysgeusia | 2 (25) | | 2 (33.3) | | 0 | | 1 (16.7) | | | 0 | | 3 (12.5) | | 5 (15.6) |

TEAE, treatment-emergent adverse event

#### **Supplementary Table S5. Pharmacokinetic results of JNJ-61393215 following multiple oral doses**

|  |  | | **MAD** | |  | |  | |
| --- | --- | --- | --- | --- | --- | --- | --- | --- |
| **PK parameters** | **5 mg JNJ-61393215** | | **15 mg JNJ-61393215** | | **45 mg JNJ-61393215** | | **90 mg JNJ-61393215** | |
|  | Day 1 | Day 7 | Day 1 | Day 7 | Day 1 | Day 7 | Day 1 | Day 7 |
| **N** | 6 | 6 | 6 | 6 | 6^a^ | 5 | 6 | 6^b^ |
| **C_max_ (ng/mL)** | 414 (73.9) | 627 (272) | 1167 (106) | 1380 (248) | 2890 (1190) | 3528 (1227) | 4575 (1261) | 4784 (1454) |
| **t_max_ (h),**  **median (range)** | 1.00  (0.67-1.50) | 1.25  (0.67-2.02) | 2.00  (0.67-3.02) | 2.00  (0.67-3.00) | 1.75  (1.00-3.00) | 1.50  (0.67-3.00) | 2.00  (1.50-4.00) | 1.50  (1.00-3.00) |
| **AUC_24h_ (ng.h/mL)** | 5726  (1715) | 8401  (5604) | 14266 (3263) | 18170 (4611) | 43413 (18665) | 48187 (17359) | 64642 (20606) | 66403 (20245) |
| **FU, 0h** | 0.0177 (0.00237) | 0.0194 (0.00413) | 0.0196 (0.00587) | 0.0234 (0.00742) | 0.0177 (0.00744) | 0.0184 (0.00517) | 0.0177 (0.00473) | 0.0226 (0.00698) |

Data are represented as mean±SD unless specified.^a^ n=5 for AUC_24h_, ^b^ n=5 for C_max_, t_max_.

AUC_24h_, area under the plasma concentration-time curve from time 0 to 24 h; C_max_, maximum plasma concentration; FU, Unbound fraction; MAD, multiple-ascending dose; t_max_, time to reach maximum plasma concentration.

### **Supplementary Figures**

#### **Supplementary Figure S1. Study design**

**
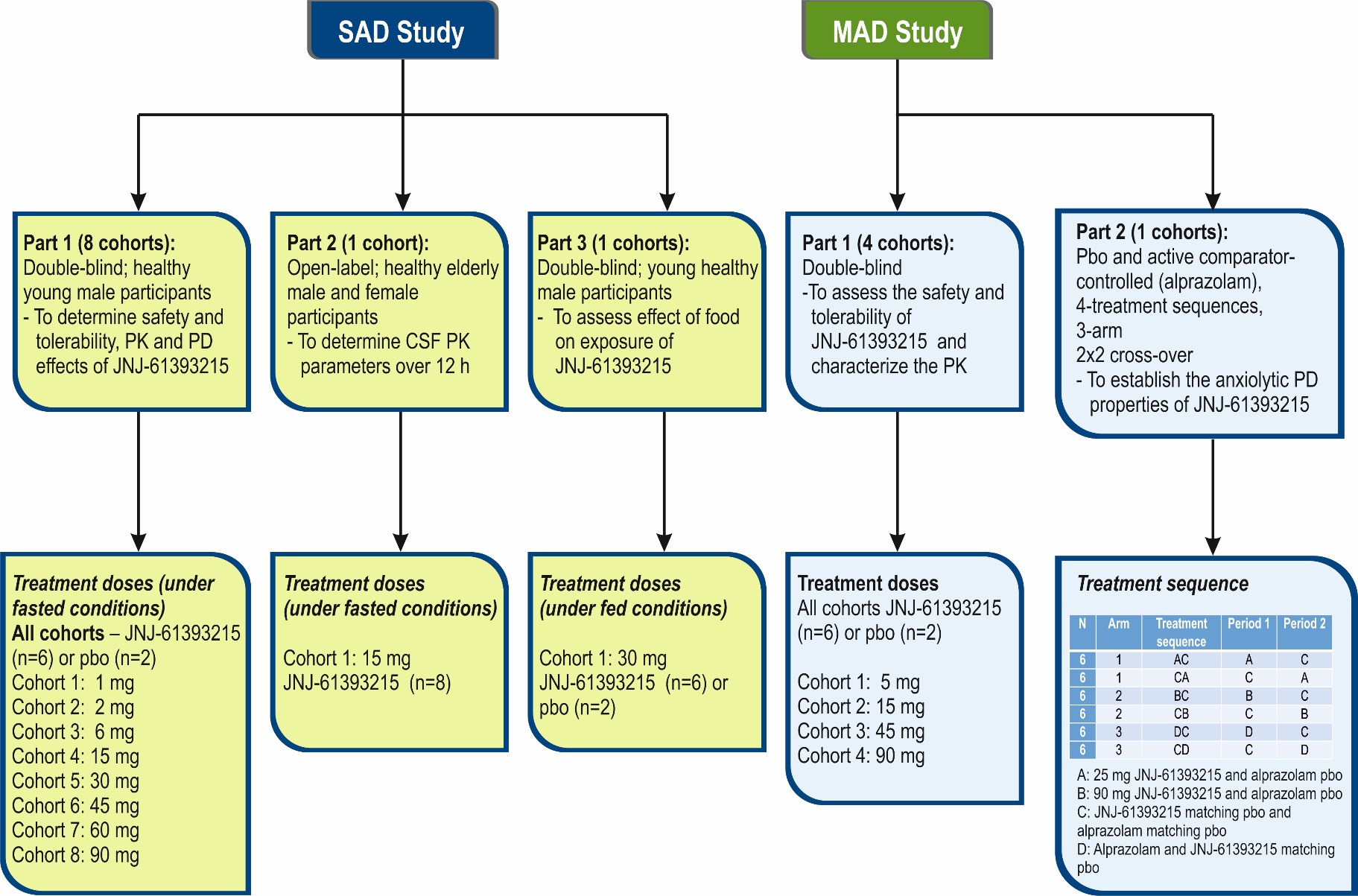
**

*Part 2 and 3 results of SAD study are not presented in this report.

MAD, multiple-ascending dose; N, number of patients; Pbo, placebo; PD, pharmacodynamics; PK, pharmacokinetic; SAD, single-ascending dose. JNJ-61393215 was administered orally QD; alprazolam 1 mg BID.

#### **Supplementary Figure S2. REM sleep-promoting effects of JNJ-61393215 in OX2R KO and OXR2 WT.**

REM sleep latency and duration in OX2R KO

A

REM sleep latency and duration in OX2R WT

B

NREM sleep latency and duration in OX2R KO

**C**

NREM sleep latency and duration in OX2R WT

**D**

REM sleep latency and duration in OX2R KO (A) and OX2R WT (B), and NREM sleep latency and duration in OX2R KO (C) and OX2R WT (D) for the 6 h period after oral dosing (30 mg/kg) during the light phase are expressed in minutes. Values are means ± S.E.M. of 9 OX2R KO and 5 OX2R WT mice. *p<0.05 and **p<0.01 versus vehicle as determined by paired Student’s t test (REM Latency). *p<0.05 versus vehicle based on two-way ANOVA (interaction Time x Treatment) followed by Bonferroni post-hoc test (REM Duration per 2 h intervals).

**Supplementary text**

In the human study, JNJ-61393215 was supplied as a clear solution containing a bittering agent denatonium benzoate 1µg/ml.

#### **Compound formulation and administration**

For animal studies, the dosing formulations were prepared in 20% hydroxypropyl-β-Cyclodextrin). In the human study, JNJ-61393215 was supplied as a clear solution containing a bittering agent denatonium benzoate 1µg/ml.

#### **Ex vivo receptor labeling**

OX1R radioligand binding autoradiography was determined at room temperature with 5 nM [^3^H]Compound 56 in 50 mM Tris HCl with 10mM MgCl2 and 5mM EDTA [^12^](#_ENREF_12). Sections were incubated for 10 min to minimize dissociation. Non-specific binding was determined in the presence of 10 μM SB-674042. Ex vivo receptor labeling was expressed as the percentage of receptor labeling in corresponding brain areas (i.e., tenia tecta) of vehicle-treated animals. The percentage of receptor occupancy was plotted against time or dosage using GraphPad Prism (GraphPad Software, San Diego, CA, United States). Percentage of receptor occupancy was also plotted against experimental treatment plasma or brain concentration. Pharmacokinetic parameters were analyzed using a non-compartmental model using the software package WinNonlin Version 4.0.1 (Pharsight, Palo Alto, CA, United States).

#### **Study population**

The enrolled participants had to be healthy and medically stable on the basis of clinical laboratory tests, and physical and neurological examination at screening. The participants who were medically stable with medication were included after assessment with written agreement of the sponsor’s responsible safety physician.

Participants with any clinically significant abnormality on neurological examination at screening or admission or any relevant history of lower back pain or scoliosis and/or major back surgery, or signs of increased intracranial pressure based on fundoscopy at screening were excluded. Participants allergic to local anesthetics and/or iodine, or had aspirin, spinal catheter insertion, or had any low molecular weight heparin or any antiplatelet drug or a topical infection or local dermatological condition at the puncture site prior to lumbar puncture and spinal catheter insertion were also excluded.

#### **Pharmacokinetic, pharmacodynamic and safety parameters**

The pharmacokinetic parameters included maximum observed concentration (C_max_), time to reach maximum observed concentration (t_max_), the area under the plasma concentration-time curve (AUC) from time 0 to time of the last observed quantifiable concentration (C_last_) calculated using linear trapezoidal rule (AUC_last_), AUC from time 0 to infinite time (AUC_∞_) and the elimination half-life (t_1/2term_) associated with terminal slope (λz, first order rate constant associated with terminal portion of curve). The total apparent systemic clearance (CL/F), apparent volume of distribution based on terminal phase (Vd/F) were calculated. C_max_, C_last_, t_max_, AUC_last_, AUC_α_, t_½ term_, CL/F, Vd/F in SAD study and and C_max_, t_max_, AUC_24h_, unbound fraction, CL/F, Vd/F in MAD study were estimated in all participants who received a dose of JNJ‑61393215. Participant safety was monitored by regular assessment of the results of clinical laboratory tests, electrocardiogram, physical examination, vital signs, and treatment-emergent adverse events (TEAEs) as reported by parents, or medical staff.

#### **Test animals: Brain and plasma samples processing**

Brain samples were rapidly frozen on powdered dry ice and stored at -80°C before sectioning. Plasma and brain samples were also collected for bioanalysis and concentrations were determined using qualified, specific and sensitive liquid chromatography-mass spectrometry/mass spectroscopy (LC–MS/MS) methods. Brain tissue samples were homogenized in water (1:3, weight/volume ratio) prior to bioanalysis. Approximately 20 µM thick tissue sections at the level of the tenia tecta were prepared for autoradiography (ex vivo labelling methodology using [^3^H] Compound56 is described in supplementary text).

#### **Protein binding assays**

The unbound fraction in brain homogenate was then scaled to the whole brain. The blood samples were collected at predose and 2.5h postdose for the determination of plasma protein-binding. The unbound fraction of ^14^C -JNJ-61393215 was determined from a single plasma protein-binding sample. Aliquots from the plasma protein-binding sample were used to determine unbound fraction via equilibrium dialysis. Samples were incubated at 37ºC for 6 h. The plasma samples from the clinical studies were fortified with ^14^C -JNJ-61393215 (230 ng/ml, 1.04 kBq/ml) before equilibrium dialysis for the in vitro determination. After dialysis, the concentration of ^14^C-JNJ-61393215 in buffer and plasma was determined using liquid scintillation counting.

#### **Sleep recording and analysis in rats and mice**

Animals were chronically implanted with telemetric devices (Data Sciences International, St. Paul, MN, United States) for the recording of electroencephalogram (EEG) and electromyogram (EMG) signals. Polysomnographic waveforms were analyzed per 10-s epoch and classified as wake, non-rapid eye movement (NREM) or REM sleep by using the computer software program SleepSign (Kissei Comtec, Nagano, Japan). For each experiment, EEG and EMG signals were recorded for up to 6 h after administration of test compounds. Analysis of sleep parameters included latency to NREM sleep (defined as the time interval to the first six consecutive NREM epochs) and REM sleep (the first two consecutive REM epochs post-treatment), and the duration of NREM and REM. Results were averaged and expressed as mean ± SEM in defined time intervals.
